# The Association Between Actigraphy-Derived Behavioral Clusters and Self-Reported Fatigue in Persons With Multiple Sclerosis: Cross-sectional Study

**DOI:** 10.2196/31164

**Published:** 2022-03-17

**Authors:** Philipp Gulde, Peter Rieckmann

**Affiliations:** 1 Center for Clinical Neuroplasticity Medical Park Loipl Medical Park Societas Europaea Bischofswiesen Germany; 2 Human Movement Science Department of Sport and Health Sciences Technical University of Munich Munich Germany

**Keywords:** multiple sclerosis, actigraphy, cluster analysis, fatigue, physical activity, neurology, neurorehabilitation, rehabilitation, digital health, health technology, digital tools

## Abstract

**Background:**

Persons with multiple sclerosis frequently report increased levels of fatigue and fatigability. However, behavioral surrogates that are strongly associated with self-reports are lacking, which limits research and treatment.

**Objective:**

The aim of this study was to derive distinct behavioral syndromes that are reflected by self-reports concerning fatigue and fatigability.

**Methods:**

We collected actigraphic data of 30 persons with multiple sclerosis over a period of 1 week during an inpatient stay at a neurorehabilitation facility. Further, participants completed the German fatigue severity scale. A principal component analysis of actigraphic parameters was performed to extract the latent component levels of behaviors that reflect fatigue (quantity of activity) and fatigability (fragmentation of activity). The resulting components were used in a cluster analysis.

**Results:**

Analyses suggested 3 clusters, one with high activity (*d*=0.65-1.57) and low clinical disability levels (*d*=0.91-1.39), one with high levels of sedentary behavior (*d*=1.06-1.58), and one with strong activity fragmentation (*d*=1.39-1.94). The cluster with high levels of sedentary behavior further revealed strong differences from the other clusters concerning participants’ reported levels of fatigue (*d*=0.99-1.28).

**Conclusions:**

Cluster analysis data proved to be feasible to meaningfully differentiate between different behavioral syndromes. Self-reports reflected the different behavioral syndromes strongly. Testing of additional domains (eg, volition or processing speed) and assessments during everyday life seem warranted to better understand the origins of reported fatigue symptomatology.

## Introduction

Persons with multiple sclerosis (MS) frequently show low levels of physical activity and increased levels of sedentary behavior [1,2] and report high levels of fatigue and fatigability [3-6]. Although fatigue is often used as an umbrella term for being exhausted in a resting state (fatigue) and easily entering a state of exhaustion (fatigability), fatigue and fatigability represent 2 different dimensions [7]. This is important since they therefore need to be assessed as 2 distinct dimensions to evaluate the progression of the disease or the effects of interventions (eg, medication or physical therapy). It has been shown that reported levels of fatigue are associated with reduced quality of life [4]. However, studies have revealed very little to no meaningful association between objectively assessed function (capacity) or behavior and self-reported dimensions like quality of life, fatigue, or depression [4,8]. Such a missing association could indicate either insufficient validity of self-reports or objective assessments or, alternatively, low sensitivity of self-reports or current approaches to objectively assess such psychological constructs. Especially when considering fatigue and fatigability (since they are commonly assessed or recognized by their consequence, which is a lack of activity), actigraphy could be a feasible measure to continuously gather objective data [9] and circumvent “assessing a snapshot of the person’s feelings and current interpretation of subjective experience” [4]. When anticipating a certain intraindividual and interindividual variance of self-reports, as there can be a plethora of biases [10-12], cluster analyses of actigraphic data would have the potential to identify behavioral patterns and validate self-reports by treating a cluster of persons as one type of person. Such an approach could allow us to extract general rules and acknowledge that humans are not very skilled in estimating global aspects of their life [11] or their current sensorimotor performance capacity [13] (ie, that humans tend to show strong interpersonal and intrapersonal variance in self-reports). However, such an exploratory cluster analysis could also result in a single cluster, for instance, if the assessed behavior is a single continuum with only one attractor. The added value of this approach would be the possibility of objectively classifying persons and further avoiding restrictions of self-reports (independent of the number of clusters) and further allowing sensitive longitudinal data collection, for instance, to test responsiveness to medication or to allow precision therapies.

Actigraphy is already a frequently used method to assess behavior in MS [14-17]. In actigraphic studies, persons with MS commonly show less activity and higher levels of sedentary behavior than healthy controls [14,16]. Further, persons with MS who have higher grades of disability, especially more strongly impaired ambulatory function, show a shift in activity intensity (eg, less moderate but more light activity) [15,16]. This approach generally reveals good psychometric properties when assessing the behavior of persons with MS [17].

In this study, we collected actigraphic and self-reported data of persons with MS to examine if behavioral patterns can help us better understand self-reports. We hypothesized that cluster analyses would reveal behavioral clusters that also show psychometric and clinical differences.

## Methods

### Participants

A convenience sample of 30 persons with MS was used for this study (Table 1). All participants were recruited during an inpatient rehabilitation stay at a specialist clinic for neurology, the Center for Clinical Neuroplasticity, Medical Park Loipl, in Germany. The following exclusion criteria were used: the inability to walk, strong depressive symptomatology (ie, Beck Depression Inventory-II scores of ≥20), other diagnosed psychiatric disorders, an age of <18 years, and the inability to give written informed consent. Regarding the clinical severity of MS, the sample showed a mean Expanded Disability Status Scale (EDSS) score of 3.5 and concerning the sensorimotor performance, a mean Watzmann Severity Scale (WSS) score of 3.5. The EDSS score was determined by trained neurologists from the specialist clinic and the WSS was assessed on first contact with the participants. Overall, the neurological status of the patients’ MS was mild to moderate. Of the 30 participants, 22 (73%) presented with relapsing-remitting MS and 8 (27%) presented with a progressive form of MS [18]. This was based on each participant’s medical records and an interview (by a trained neurologist on rehabilitation entry) on the course of MS for confirmation. The mean disease duration, taken as the time since the patient’s first diagnosis, was between 0 and 24 years, with a mean of 7.5 years (Table 1). Demographic and clinical characteristics were not only taken into account for comparability, but also considered due to mixed reports on their association with self-reported fatigue [19,20].

**Table 1:** Demographic and clinical characteristics of the participants (N=30).

Characteristic		Value
Age in years, mean (SD), range		43.7 (11.5), 21-65
**Sex, n (%)**		
	Female	19 (63)
	Male	11 (37)
BMI in kg/m², mean (SD), range		26.2 (4.7), 18.2-35.1
Expanded Disability Status Scale score, mean (SD), range		3.5 (1.4), 1.0-6.5
Watzmann Severity Scale score, mean (SD), range		3.5 (1.1), 1.7-5.8
**Type of multiple sclerosis, n (%)**		
	Relapsing-remitting	22 (73)
	Progressive	8 (27)
Time since first diagnosis (DISDUR) in years, mean (SD), range		7.5 (6.6), 0-24

### Ethics Approval

Ethical approval was granted by the ethics committee of the medical faculty of the Technical University of Munich on July 14, 2020 (approval identifier: 478/19 S-SR). All participants provided written informed consent.

### Study Parameters

Each participant was asked to wear a wrist-worn actigraph (ActiGraph wGT3X-BT, ActiGraph LLC; the 100 Hz measurement frequency was downsampled to 1 Hz, ie, 1-second epochs) on the dominant or better functioning side (concerning the upper limb) of their body for 1 full week. The triaxial acceleration signal was collected and stored as compressed raw data on the device. After that period, participants completed the German fatigue severity scale (FATIGUE parameter) [21] to assess their experienced level of fatigue. The parameters that were extracted from the actigraphic data were the average number of daily steps (STEPS), the body mass–adjusted metabolic equivalent (MET), the estimated ratio of sedentary behavior (SEDENTARY), and the ratio of the number of activity bouts lasting ≥5 minutes and ≥10 minutes (RATIO) [22]. STEPS aimed to assess kinematic physical activity, MET assessed dynamometric physical activity, SEDENTARY was a coarse estimate of fatigue, and RATIO assessed fatigability. The metabolic equivalent and time in sedentary behavior were estimated by the actigraph (using ActiLife software, version 6.13.4; ActiGraph LLC); the ActiLife software was based on the Freedson adult algorithm [23]. The threshold used for sedentary behavior was 99 activity counts per minute.

A 2-component confirmatory principal component analysis with a varimax rotation for the actigraphic data was calculated (1 component as fatigue and 1 as fatigability). Thresholds for the Kaiser-Meyer-Olkin test of sample adequacy were set to ≥0.50, and minimum communalities were set to ≥0.50. Based on the component scores, a cluster analysis using k-means clustering (Hartigan-Wong) was performed. The number of clusters was determined from a scree plot. Cluster differences in terms of behavioral, psychometric, and demographic or clinical properties were tested by analyses of variance; for sex and type of MS, chi-square tests were used. Effect-sizes were derived post hoc using the Cohen *d*. α was set to .05. All statistical tests were run using R (version 1.4.1106; R Foundation for Statistical Computing).

## Results

**Figure 1:**
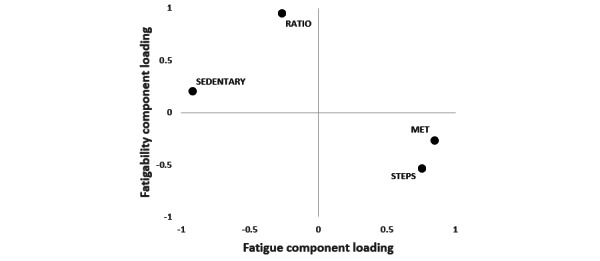
Component loadings of the 4 different actigraphic parameters. MET: body mass–adjusted metabolic equivalent. SEDENTARY: estimated ratio of sedentary behavior. STEPS: number of steps per day. RATIO: ratio of the number of activity bouts lasting ≥5 minutes and ≥10 minutes.

The actigraphic and psychometric outcomes for the sample are reported in Table 2. There were no missing data; the actigraphs were tolerated during nighttime and were waterproof, so participants had 7 complete 24-hour data sets. The overall measure of sample adequacy was middling, with 0.75, and none of the 4 parameters scored below 0.50 (Table 2). The principal component analyses had a proportion of explained variance of 0.88 and communalities of 0.80 for MET, 0.86 for STEPS, 0.88 for SEDENTARY, and 0.98 for RATIO. The component loadings are displayed in Figure 1. Component 1 (Figure 1, x-axis) correlated with FATIGUE (*r*=–0.54; *P*=.002), but not component 2 (Figure 1, y-axis; *r*=–0.13; *P*=.49).

**Figure 2:**
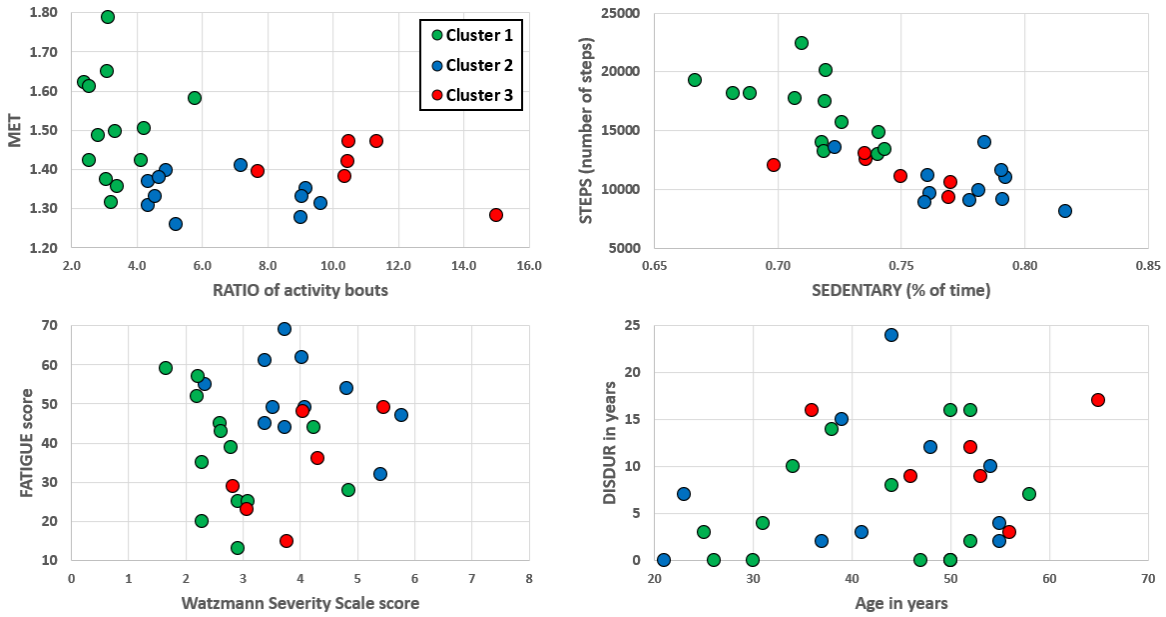
Illustration of cluster distributions in different parameter pairs. MET: body mass–adjusted metabolic equivalent. RATIO: ratio of the number of activity bouts lasting ≥5 minutes and ≥10 minutes. STEPS: number of steps per day. SEDENTARY: estimated ratio of sedentary behavior. FATIGUE: German fatigue severity scale score. DISDUR: time since initial diagnosis.

The cluster analysis resulted in 3 clusters; cluster 1 had 11 persons, cluster 2 had 13 persons, and cluster 3 had 6 persons. Table 3 reports the statistical differences between the resulting clusters. Cluster 1 had higher STEPS and MET and lower RATIO, SEDENTARY, EDSS, and WSS than the other 2 clusters. Cluster 2 showed the highest SEDENTARY and FATIGUE values and cluster 3 had the highest RATIO values. Clusters 2 and 3 were similar in terms of STEPS, MET, EDSS, and WSS. All clusters were comparable concerning the following variables: age, sex distribution, BMI, DISDUR (time since diagnosis), and type of MS (Table 3). As with component 1 (Fatigue component), MET, STEPS, and SEDENTARY were significantly associated with FATIGUE, while RATIO showed no significant correlation with FATIGUE (Table 4). Figure 2 illustrates the individual scores of persons in the different clusters for key parameters like the WSS score.

**Table 2:** Actigraphic and psychometric outcomes among participants.

Parameter	Mean (SD)	Range	Measure of sample adequacy
STEPS^a^	13,400 (3800)	8100-22,400	0.71
MET^b^	1.43 (0.11)	1.26-1.76	0.85
RATIO^c^	6.0 (3.4)	2.4-15	0.74
SEDENTARY^d^	0.74 (0.04)	0.67-0.82	0.71
FATIGUE^e^	41.7 (14.6)	13-69	0.74

^a^STEPS: number of steps per day.

^b^MET: body mass–adjusted metabolic equivalent.

^c^RATIO: ratio of the number of activity bouts lasting ≥5 minutes and ≥10 minutes.

^d^SEDENTARY: estimated ratio of sedentary behavior.

^e^FATIGUE: German fatigue severity scale score.

**Table 3:** Cluster comparisons including means, SDs, and ranges.

Parameter			Cluster 1		Cluster 2		Cluster 3		*P* value^a^		Post hoc comparisons
STEPS^b^, mean (SD), range			16,700 (3000), 12,900-22,400		10,500 (1900), 8100-14,000		11,400 (1400), 9300-13,100		<.001		Cluster 1-2: *P*<.001; *d*=0.99 Cluster 1 and 3: *P*<.001; *d*=0.65
MET^c^, mean (SD), range			1.53 (0.09), 1.44-1.76		1.34 (0.05), 1.26-1.41		1.40 (0.07), 1.28-1.47		<.001		Cluster 1-2: *P*<.001; *d*=1.57 Cluster 1-3: *P*=.007; *d*=1.22
RATIO^d^, mean (SD), range			3.4 (0.9), 2.4-5.8		6.6 (2.3), 4.3-9.6		10.9 (2.4), 7.7-15.0		<.001		Cluster 1-2: *P*<.001; *d*=–1.39 Cluster 1 and 3: *P*<.001; *d*=–1.94 Cluster 2-3: *P*<.001; *d*=–1.41
SEDENTARY^e^, mean (SD), range			0.71 (0.02), 0.67-0.74		0.78 (0.02), 0.72-0.82		0.74 (0.03), 0.70-.077		<.001		Cluster 1-2: *P*<.001; *d*=–1.58 Cluster 1-3: *P*=.05; *d*=–1.06 Cluster 2-3: *P*=.03; *d*=1.13
FATIGUE^f^, mean (SD), range			37.3 (14.4), 13-59		51.5 (10.2), 32-69		33.3 (13.6), 15-49		.01		Cluster 1-2: *P*=.01; *d*=–0.99 Cluster 2-3: *P*=.02; *d*=1.28
Age in years, mean (SD), range			41.3 (11.2), 25-58		42.5 (11.9), 21-55		51.3 (9.8), 36-65		.19		N/A^i^
**Sex, n (%)**											
	Female	8 (77)		8 (64)		2 (33)		.19		N/A	
	Male	3 (23)		5 (36)		4 (67)		.19		N/A	
BMI in kg/m², mean (SD), range			25.8 (4.0), 20.9-32.2		27.0 (4.8), 18.2-33.8		25.6 (6.4), 18.7-35.1		.80		N/A
Expanded Disability Status Scale score, mean (SD), range			2.7 (1.1), 1.0-5.0		3.9 (1.4), 2.0-6.5		4.7 (1.1), 3.5-6.5		.005		Cluster 1-2: *P*=.03; *d*=–0.91 Cluster 1-3: *P*=.005; *d*=–1.39
Watzmann Severity Scale score, mean (SD), range			2.8 (0.9), 1.7-4.9		4.0 (1.0), 2.3-5.8		3.9 (0.9), 2.8-5.5		.008		Cluster 1-2: *P*=.005; *d*=–1.10 Cluster 1-3: *P*=.04; *d*=–1.08
DISDUR^g^ in years, mean (SD), range			6.2 (6.2), 0-16		7.2 (7.5), 0-24		11.0 (5.2), 3-17		.33		N/A
**TYPE^h^, n (%)**											
	Relapsing-remitting	8 (77)		9 (73)		4 (67)		.89		N/A	
	Progressive	3 (23)		4 (27)		2 (33)		.89		N/A	

^a^The *P* values for all parameters, except sex and TYPE, were derived using ANOVA. The *P* values for sex and TYPE were derived using the chi-square test.

^b^STEPS: number of steps per day.

^c^MET: body mass–adjusted metabolic equivalent.

^d^RATIO: ratio of the number of activity bouts lasting ≥5 minutes and ≥10 minutes.

^e^SEDENTARY: estimated ratio of sedentary behavior.

^f^FATIGUE: German fatigue severity scale score.

^g^DISDUR: time since initial diagnosis.

^h^TYPE: type of multiple sclerosis.

^i^N/A: not applicable.

**Table 4:** The correlation between FATIGUE and demographic, clinical, and actigraphic parameters.

Variable			Age		BMI		EDSS^b^		WSS^c^		DISDUR^d^		MET^e^		STEPS^f^		SEDENTARY^g^		RATIO^h^
**FATIGUE^a^**																			
	*r*	–0.03		0.22		0.23		0.04		–0.0003592		–0.43		–0.41		0.43		0.04	
	*P* value	.87		.25		.22		.83		.99		.02		.03		.02		.98	

^a^FATIGUE: German fatigue severity scale score.

^b^EDSS: Expanded Disability Status Scale score.

^c^WSS: Watzmann Severity Scale score.

^d^DISDUR: time since initial diagnosis.

^e^MET: body mass–adjusted metabolic equivalent.

^f^STEPS: number of steps per day.

^g^SEDENTARY: estimated ratio of sedentary behavior.

^h^RATIO: ratio between short and longer activity bouts.

## Discussion

### Principal Findings

In this study, we assessed data on physical activity and reported levels of fatigue and depression from 30 persons with MS during an inpatient stay at a rehabilitation facility. Although not being in the home environment, behavioral characteristics could be similar [24]. Statistical modelling confirmed our initial differentiation of parameters surrogating fatigue (SEDENTARY) and fatigability (RATIO) and suggested 3 clusters, which revealed very strong differences in actigraphic parameters, reported levels of fatigue, and the severity of MS (clinical and sensorimotor). Cluster 1 was the most active group with more daily steps (STEPS), higher body mass–adjusted metabolic equivalents (MET), smaller ratios between short and longer activity bouts (RATIO), less sedentary behavior (SEDENTARY), and lower EDSS and WSS scores than the other 2 clusters. Cluster 2 showed the highest ratios of sedentary behavior (SEDENTARY) and reported the highest levels of fatigue (FATIGUE). Cluster 3 had the highest ratios between short and longer activity bouts (RATIO). Overall, there was 1 active cluster with the lowest disability (cluster 1), 1 cluster with the highest signs of and reported fatigue (cluster 2), and 1 cluster with the highest fatigability (cluster 3). Clusters 2 and 3 had comparable MET and STEPS as well as clinical and sensorimotor disease severity, and clusters 1 and 3 had comparable levels of reported fatigue. This is in line with other studies that showed reported fatigue to be quite independent of performance [25] since our fatigue cluster revealed intermediate ratios of short and longer activity bouts (RATIO). The cluster with higher fatigability, on the contrary, reported average levels of fatigue (FATIGUE). Interestingly, the differentiation between the various behavioral clusters had strong effect-sizes, while correlations were quite weak, which supports our initial thoughts that led to the clustering approach (ie, low reliability of persons assessing their own condition, which can be circumvented by objective sensor-supported assessments). This would also allow for the monitoring of the psychological and behavioral course of persons with MS (or, for instance, frail elderly individuals, stroke survivors, etc.) in a reliable and valid way. Such information, of course, needs to be understood as a complementary, not alternative, data source. However, it is important to note that all clusters had individuals reporting very high levels of fatigue, which is important concerning the validity of the used questionnaire, as this has been questioned for a set of fatigue questionnaires in general [26]. Interestingly, there were no significant differences between the clusters concerning most of the demographic and clinical characteristics such as TYPE (type of MS), DISDUR (time since initial diagnosis), BMI, age, or sex; however, there was a significant difference between the clinical and sensorimotor severity of the condition. This suggests that the assessed dimensions were not strongly influenced by conceivable confounders like BMI, age, or biological sex and that fatigue and fatigability could be seen as valid psychological constructs. Further, none of the nonactigraphic parameters were associated with the reported levels of fatigue. As shown in other publications on the topic [19,20], the outcomes concerning the associations of self-reported fatigue and demographic and clinical characteristics can strongly depend on the statistical approach used, underscoring the need to employ objective assessments like, in our case, actigraphy to overcome the limited reliability of self-reports [4,13].

### Limitations

It is crucial to note that the interpretation of our findings is based on the assumption that fatigability leads to more fragmented activity, but not necessarily less volume of activity. Concerning the potentially limited validity of the questionnaire used, the following factor may have been involved: a bias towards extremes within the questionnaire (none of the single items were normally distributed, but the sum score of the questionnaire was) due to humans being quite inaccurate in estimating their own conditions and differentiating between state and trait [11]. Further, low item difficulties can prevent the identification of persons with extremely high levels of fatigue [27].

### Conclusions

To conclude, clustering of behavioral data proved to be a strong approach in examining self-reports. Our analyses, suggesting 3 different clusters, deliver behavioral correlates of the fatigue and fatigability constructs and warrant future studies on actigraphy in the home environment of persons with MS. A further examination of the feeling of fatigue by objective psychometric means (eg, tests of problem-solving, motivational priming, processing speed measured by reaction time) would be recommended to better understand if the umbrella term of fatigue dominantly arises from bodily, cognitive, or emotional domains [28].

## Abbreviations

EDSS: Expanded Disability Status Scale
MS: multiple sclerosis
WSS: Watzmann Severity Scale
